# Malnutrition is associated with six-month mortality in older patients admitted to the emergency department with hip fracture

**DOI:** 10.3389/fmed.2023.1173528

**Published:** 2023-04-20

**Authors:** Kristina Franz, Johannes Deutschbein, Dorothee Riedlinger, Mareen Pigorsch, Liane Schenk, Tobias Lindner, Martin Möckel, Kristina Norman, Ursula Müller-Werdan

**Affiliations:** ^1^Charité – Universitätsmedizin Berlin, Corporate Member of Freie Universität Berlin and Humboldt-Universität zu Berlin, Department of Geriatrics and Medical Gerontology, Geriatrics Research Group, Berlin, Germany; ^2^Charité – Universitätsmedizin Berlin, Corporate Member of Freie Universität Berlin and Humboldt-Universität zu Berlin, Institute of Medical Sociology and Rehabilitation Science, Berlin, Germany; ^3^Charité – Universitätsmedizin Berlin, Corporate Member of Freie Universität Berlin and Humboldt-Universität zu Berlin, Division of Emergency Medicine Campus Mitte and Virchow, Berlin, Germany; ^4^Charité – Universitätsmedizin Berlin, Corporate Member of Freie Universität Berlin and Humboldt-Universität zu Berlin, Institute of Biometry and Clinical Epidemiology, Berlin, Germany

**Keywords:** hip fracture, malnutrition, acute medicine, emergency department, geriatrics, short nutritional assessment questionnaire, health services research

## Abstract

**Background:**

Hip fractures in older people are a common health problem often associated with malnutrition that might affect outcomes. Screening for malnutrition is not a routine examination in emergency departments (ED). This analysis of the EMAAge study, a prospective, multicenter cohort study, aimed to evaluate the nutritional status of older patients (≥ 50 years) with hip fracture, factors associated with malnutrition risk, and the association between malnutrition and the six-months mortality.

**Methods:**

Risk of malnutrition was evaluated using the Short Nutritional Assessment Questionnaire. Clinical data as well as data on depression and physical activity were determined. Mortality was captured for the first six months after the event. To assess factors associated with malnutrition risk we used a binary logistic regression. A Cox proportional hazards model was used to assess the association of malnutrition risk with six-month survival adjusted for other relevant risk factors.

**Results:**

The sample consisted of *N* = 318 hip fracture patients aged 50 to 98 (68% women). The prevalence of malnutrition risk was 25.3% (*n* = 76) at the time of injury. There were no differences in triage categories or routine parameters measured in the ED that could point to malnutrition. 89% of the patients (*n* = 267) survived for six months. The mean survival time was longer in those without malnutrition risk (171.9 (167.1–176.9) days vs. 153.1 (140.0–166.2) days). The Kaplan Meier curves and the unadjusted Cox regression (Hazard Ratio (HR) 3.08 (1.61–5.91)) showed differences between patients with and patients without malnutrition risk. In the adjusted Cox regression model, risk of death was associated with malnutrition risk (HR 2.61, 1.34–5.06), older age (70–76 years: HR 2.5 (0.52–11.99); 77–82 years: HR 4.25 (1.15–15.62); 83–99 years: HR 3.82 (1.05–13.88)) and a high burden of comorbidities (Charlson Comorbidity Index ≥3: HR 5.4 (1.53–19.12)).

**Conclusion:**

Risk of malnutrition was associated with higher mortality after hip fracture. ED parameters did not differentiate between patients with nutritional deficiencies and those without. Therefore, it is particularly important to pay attention to malnutrition in EDs to detect patients at risk of adverse outcomes and to initiate early interventions.

## Introduction

Hip fractures are a common musculoskeletal injury in older people with a serious impact on the patient’s daily life. Consequences are reduced mobility, loss of independence, impaired quality of life, and increased morbidity (1). Patients with hip fracture make up a significant proportion of the growing number of older and multimorbid adults with complex medical and psychosocial problems, posing a challenge to healthcare providers, including emergency departments (2). Malnutrition is a particularly pronounced health problem in old age and is associated with a higher risk of hip fracture in older adults (3).

Old adults are particularly prone to nutritional deficiencies due to their age-associated physiological changes and decreasing physiological reserves (4), thus they are of high risk to develop malnutrition (5). Causes of malnutrition in older adults are multifactorial and associated with physiological, pathological, social, and mental problems in old age (6). A systematic review of the literature based on longitudinal data revealed significant risk factors for malnutrition in older adults such as age, polypharmacy, impairments in physical function, cognitive impairment, dementia, loss of appetite and poor nutritional status, depressive symptoms as well as institutionalization (7). Patients with hip fracture have a considerably lower intake of calories and protein during the first 10 days of hospitalization than recommended (8). Furthermore, the increase in energy demand due to inflammation results in a catabolic status that persists for up to 4 months after the fracture (9, 10). Following weight loss, older adults are less able to regain body weight, particularly lean mass, compared to younger adults (11). This is attributed to a lack of adaptation in energy metabolism as well as reduced postprandial muscle protein synthesis (12, 13). Malnutrition has a significant negative impact on functional status and leads to reduced rehabilitation rates in hip fracture patients (14). The health economic importance of malnutrition in patients with hip fracture results from longer inpatient stays increased rehospitalization rates, and increased resource consumption and treatment costs (15, 16). This emphasizes the need for nutritional support in clinical (17) as well as in community and long-term care settings (18).

Few studies have addressed malnutrition in older patients in emergency departments (ED), and especially in Germany there is too little data on the prevalence of malnutrition in ED patients with hip fractures and its correlation with mortality. Previous studies have reported a prevalence of malnutrition in older ED patients up to 29% (19–22) which was associated with a higher mortality (20, 23).

The aim of this study was to determine the prevalence of self-reported signs of malnutrition in a cohort of older ED patients with hip fracture (≥ 50 years), to explore factors associated with risk of malnutrition, and to analyze the association between risk of malnutrition and six-month mortality.

## Materials and methods

### Study design and study population

Analyzes are based on data from the EMAAge study. EMAAge is a multicenter, prospective cohort study in the context of EMANet, a regional network of health services research in emergency and acute medicine in Berlin-Mitte, Germany (24, 25). The study included *N* = 318 patients aged 50 years and older (*M* = 76.6, SD = 11.0) with a hip fracture admitted to the EDs of six hospitals between June 2017 and June 2019 (24). Patients with dementia were included *via* their relatives or legal guardians. Participants or their proxies were interviewed by study nurses during the first days after initial treatment of the fracture by using a standardized questionnaire. Follow-up interviews were conducted *via* telephone or postal questionnaires six months later. Patient-reported data were complemented by clinical data on the ED and in-hospital care from hospital information systems. More details on the study design and data collection have been reported previously (26).

The study was approved by the ethics committee of Charité –Universitätsmedizin Berlin (EA1/362/16) and was registered in the German Clinical Trials Register (DRKS-ID: DRKS00014273). Written informed consent was obtained from all study participants. The authors confirm that all methods were carried out in accordance with good clinical practice.

### Data and variables

The following clinical parameters were extracted from the hospital information systems using an electronic clinical report form: Manchester Triage System category (MTS, categorized in high acuity: MTS levels 1 to 3 and low acuity: MTS levels 4 and 5), vital signs (systolic blood pressure, heart rate, respiratory rate and numerical pain scale), Glasgow Coma Scale (GCS) (27), ICD diagnoses, type of surgery, ICU stay, complications (yes/no), and length of hospital stay (LOS). Documented comorbidities were used to calculate the Charlson Cormorbidity Index(CCI) (28). Polypharmacy was defined as five or more medications per day. Pre-fracture care dependency was determined according to the German long-term care insurance which classifies the level of dependency in patients’ activities of daily living.

The following variables were constructed by using self-reported patient data: The standardized questionnaire the *Short Nutritional Assessment Questionnaire* (SNAQ) was used to screen for the risk of malnutrition (29). It includes the following questions: *“(1.) Did you lose weight unintentionally? More than 6 kg in the last 6 months (3 points) or More than 3 kg in the last month (2 points)? (2.) Did you experience a decreased appetite over the last month? (1 point) (3.) Did you use supplemental drinks or tube feeding over the last month? (1 point).”* The SNAQ instrument classifies patients into three groups: normal nutritional status (0–1 points), moderate risk of malnutrition (2 points) and severe risk of malnutrition (≥ 3 points).

Self-reported weight and height were used to calculate the body mass index (BMI, kg / m^2^) by using the formula BMI = body weight / height^2^. Physical activity before the fracture was assessed with the question: *“How often do you do things that are slightly or moderately strenuous?”* (≥ 1x / week, 1x / week, 1x -3x / month or never). Furthermore, participants were asked to report the frequency of previous falls (six months before the fracture). The Patient Health Questionnaire 4 (PHQ-4) was used to screen for symptoms of depression and anxiety (30). The PHQ-4 ranges from 0–12 points, increased PHQ-4 scores (cut-off ≥6) indicate depressive disorder. Cognitive impairment was assessed according to the 6-CIT screening instrument (31). Signs of moderate and severe impairment were interpreted as cognitive impairment. Patients participating *via* proxies were classified as cognitively impaired as well.

At six months, a follow-up interview was conducted. Additionally, mortality was determined through inquiries with registration offices.

### Data analysis

Descriptive parameters are given as absolute and relative frequencies (*n*, %). For continuous variables, with means and standard deviations (SD) or median and interquartile range (IQR) are reported. Effect sizes of group differences are given as standardized mean differences.

To assess factors associated with the risk of malnutrition, binary logistic regression analysis was performed. Based on a systematic review on risk factors for malnutrition (7), we included age, gender, BMI, cognitive impairment (binary category), PHQ-4 depression (binary category), the burden of comorbidities (CCI), and reduced physical activity in the binary logistic regression model as independent variables. Results are reported as odds ratios (OR) with 95% confidence intervals (CI).

The influence of malnutrition risk on the risk of mortality within the first six months after hip fracture was determined by using a Cox proportional hazards model calculating hazard ratios (HR) with 95% CI. The model was adjusted for age, gender and CCI score. In addition, Kaplan–Meier survival curves were created for patients with and without the risk of malnutrition. Survival time was calculated as the number of days from initial hospital stay to six-months follow up.

For all analyses, risk of malnutrition was used as a binary variable combining moderate and severe risk. For the multiple models, age was categorized into four groups: 50–69 years; 70–76 years; 77–82 years and 83–99 years.

To handle missing values within the regression models, we used multiple imputation (32). The imputation models consisted of the variables in the corresponding analysis model. In the imputation model for the logistic regression, we added further variables from the EMAAge study with the potential to improve the estimation of imputed data: we used items from the health-related quality of life questionnaire *EQ-5D-5L* (mobility, usual activities and anxiety (33), educational status, dependency in ADLs), the presence of a wheelchair or walker, and the usage of insoles due to urinary incontinence. For the imputation of the malnutrition variable, we first imputed the three SNAQ-variables and did a passive imputation when only single malnutrition items were missing (32). Sensitivity analyses were done for different modifications of the imputation models. For both regression models we used the R package “mice for multiple imputation” with *m* = 20 imputations (34).

The analyses of this paper are explorative and are not designed to draw confirmatory conclusions, therefore we did not correct for multiple testing. Descriptive statistics were performed using the statistics program IBM SPSS statistic (version 27). The models were done using *R* version 4.1.1.

## Results

### Characteristics of the study population

The characteristics of the study population are shown in Table 1. The analysis included *N* = 318 hip fracture patients aged 50 to 98 years (*M* = 76.6, SD = 11.0), 68% were women. For *n* = 300 participants, complete information on their risk of malnutrition could be obtained according to the SNAQ instrument. An involuntary weight loss of more than 6 kg within six months or more than 3 kg in the previous month was found in 18% (*n* = 54) and 6.7% (*n* = 20) of the patients, respectively. A quarter of the patients reported a loss of appetite in the previous month. Oral nutritional supplements were used by 6.3% (*n* = 19) of the patients. According to the SNAQ score, 22.3% (*n* = 67) of the study cohort showed a severe risk of malnutrition, followed by 3% (*n* = 9) with a moderate malnutrition risk. Compared to well-nourished patients, patients with risk of malnourishment were older and had a lower BMI (Table 1): 7.1% (*n* = 19) showed a BMI below 20 kg / m^2^ (≤ 70 years) and 20.8% (*n* = 56) below 22 kg / m^2^ (> 70 years).

**Table 1:** Characteristics of ED patients with hip fracture screened for malnutrition.

Characteristics	All (*n* = 300)	Risk of malnutrition (*n* = 76)	No risk of malnutrition (*n* = 224)	Standardized mean difference between groups
Gender (male) *n* (%)	96 (32.0)	22 (28.9)	74 (33.0)	0.097
Age (years) mean (SD)	76.6 (11.0)	79.2 (10.5)	75.7 (11.1)	0.306
*Age categories n* (%)				
50–69 years	84 (28.0)	13 (17.1)	71 (31.7)	0.376
70–76 years	37 (12.3)	10 (13.2)	27 (12.1)	
77–82 years	83 (27.7)	20 (26.3)	63 (28.1)	
83–99 years	96 (32.0)	33 (43.4)	63 (28.1)	
MTS Triage level^a^ *n* (%)				0.312
High acuity (MTS level 1–3)	212 (86.9)	62 (92.5)	150 (84.7)	
Low acuity (MTS level 4 and 5)	32 (13.1)	5 (7.5)	27 (15.3)	
Type of fracture *n* (%)				0.364
Femoral neck fractures (ICD-10 S72.0)	137 (45.7)	26 (34.2)	111 (49.6)	
Pertrochanteric fractures (ICD-10 S72.1)	138 (46.0)	44 (57.9)	94 (42.0)	
Subtrochanteric fractures (ICD-10 S72.2)	20 (6.7)	4 (5.3)	16 (7.1)	
Periprosthetic hip fractures	5 (1.7)	2 (2.6)	3 (1.3)	
BMI (kg/m^2^)^b^ mean (SD)	24.1 ± 13.9	22.5 ± 4.6	25.1 ± 5.5	0.468
*Dimensions of Malnutrition (SNAQ)*				
Involuntary weight loss *n* (%) > 6 kg within six months	54 (18.0)	54 (71.1)	–	
> 3 kg in the last month	20 (6.7)	20 (26.3)		
Loss of appetite *n* (%)	75 (25)	44 (57.9)	31 (13.8)	1.034
Intake of ONS in last three month (yes) *n* (%)	19 (6.3)	14 (18.4)	5 (2.2)	0.552
*Vital signs at ED admission median (IQR)*				
GCS (points)^c^	15 (15;15)	15 (15;15)	15 (15;15)	0.165
Systolic blood pressure (mmHg)^d^	150 (131; 161)	142 (120;157)	150 (134;163)	0.461
Heart rate (Bpm)^e^	81 (70; 90)	81 (65;89)	82 (71;90)	0.099
Respiratory rate (Breaths/min)^f^	15 (14; 17)	16 (14;17)	15 (14;17)	0.020
Numerical pain scale (points)^g^	5 (4; 6)	5 (4;7)	5 (4;6)	0.217
Polypharmacy (yes) *n* (%)	161 (53.7)	45 (59.2)	116 (51.8)	0.161
Long-term care dependency at admission (yes)^h^ *n* (%)	113 (38.7)	38 (52.1)	75 (34.2)	0.366
Admitted to ICU (yes) *n* (%)	104 (34.8)	33 (43.4)	71 (31.8)	0.229
Complications (yes) *n* (%)	167 (57.4)	51 (69.9)	116 (53.2)	0.342
LOS (length of stay in days)^i^ mean (SD)	11.7 (10.0)	13.2 (13.2)	11.0 (8.3)	0.198
*Burden of comorbidity (CCI) n (%)*				
0	87 (29.0)	15 (19.7)	72 (32.1)	0.306
1–2	118 (39.3)	32 (42.1)	86 (38.4)	
3–4	64 (21.3)	19 (25.0)	45 (20.1)	
>5	31 (10.3)	10 (13.2)	21 (9.4)	
*Cognitive impairment^j^n (%)*				
No cognitive impairment	189 (63.9)	34 (45.9)	155 (69.8)	0.483
Moderate and severe cognitive impairment	107 (36.1)	40 (54.1)	67 (30.2)	
*Depression (PHQ-4 category)*^*k*^ *n (%)*				
Depression (≥ 6 points)	32 (13.3)	12 (23.1)	20 (10.6)	0.327
No depression (< 6 points)	208 (86.7)	40 (76.9)	168 (89.4)	
*Physical activity level ^l^ n (%)*				
≥ 1x / week	155 (62.0)	20 (37.0)	135 (68.9)	0.706
1x / week	21 (8.4)	5 (9.3)	16 (8.2)	
1x - 3x / month	10 (4.0)	4 (7.4)	6 (3.1)	
Never	64 (25.6)	25 (46.3)	39 (19.9)	
*Number of falls in six months prior to hip fracture^m^n (%)*				
1	42 (46.2)	11 (39.3)	31 (49.2)	0.294
2–3	31 (34.1)	10 (35.7)	21 (33.3)	
≥ 3	18 (19.8)	7 (25.0)	11 (17.5)	
*Mortality n (%)*				
Initial hospital stay	5 (1.7)	1 (1.3)	4 (1.8)	0.040
Six-month follow up	33 (11.2)	18 (24.0)	15 (6.8)	0.431

BMI, Body Mass Index; ONS: oral nutritional supplements. ED, Emergency Department. GCS, Glasgow Coma Scale. MTS, Manchester Triage System. ICU, Intensive care unit. CCI: Charlson Comorbidity Index. Sample size of the cohort regarding each parameter: ^a^*n* = 243; ^b^*n* = 269; ^c^*n* = 257; ^d^*n* = 245; ^e^*n* = 229; ^f^*n* = 179; ^g^*n* = 166; ^h^*n* = 292; ^i^*n* = 291; ^j^*n* = 294; ^k^*n* = 240; ^l^*n* = 250; ^m^*n* = 286. ^k,l^: all proxy cases are missing by design, because these questions could only be answered by the participants themselves.

At ED admission, almost nine in 10 patients had a high acuity (1–3) in the Manchester Triage System (MTS) rating. There was no difference between patients with and without malnutrition. The vital signs at admission, namely systolic blood pressure, pain score, and GCS did not differ between patients with and without malnutrition risk. During their hospital stay, patients with malnutrition risk experienced more often complications (69.9% vs. 53.2%) and were admitted to the ICU more frequently (43.4% vs. 31.8%). Patients at risk received a higher number of medications per day (6.7 ± 4.1 vs. 5.0 ± 4.1) and were more frequently cognitively impaired (54.1% vs. 30.2%). Participants at risk of malnutrition reported more often previous falls in the last six months (36.8% vs. 28.1%). Symptoms of depression were reported by 23.1% of the patients at risk of malnutrition compared to 10.6% in the normal group. Patients at risk of malnutrition were more frequently dependent on long-term care at admission (52.1% vs. 34.2%) and had a lower physical activity level (no activity: 46.3% vs. 19.9%). During the initial hospital stay, 1.7% of all participants died, overall mortality after six months was 11.2% (Table 1).

### Parameters associated with risk of malnutrition in hip fracture patients and six-month mortality

**Figure 1:**
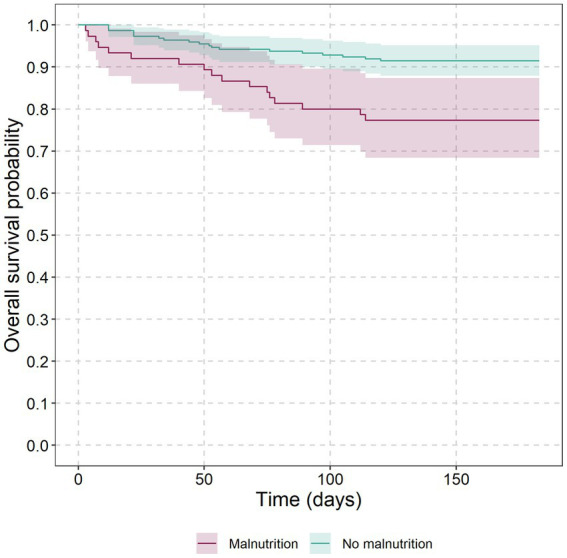
Kaplan-Meier curves for older ED patients with and without malnutrition from the beginning of the initial hospital stay after ED until the follow up at six months without adjustment. Number of patients at risk and patients who died at selected time points for malnutrition: 0 days (75 vs. 0), 50 days (68 vs. 8), 100 days (60 vs. 15) and 150 days (58 vs. 17); no malnutrition: 0 days (223 vs. 0), 50 days (213 vs. 10), 100 days (207 vs. 16) and 150 days (204 vs. 19).

Table 2 shows the results of the binary logistic regression for risk of malnutrition in ED patients with hip fracture. Higher BMI was associated with lower risk of malnutrition in ED patients (OR: 0.89, 0.83–0.96). There was a negative association between reduced physical activities and risk of malnutrition (OR: 0.28, 0.14–0.58). Risk of mortality was higher in patients with malnutrition risk (unadjusted Cox regression HR: 3.08, 1.61–5.91). Figure 1 shows the Kaplan–Meier curves indicating differences between patients with and without malnutrition risk during the first six months after hip fracture. The mean survival time was longer in patients without malnutrition risk (171.9 days; 95% CI: 167.1–176.9 days vs. 153.1 days; 95% CI: 140.0–166.2 days). In Table 3, the results of the Cox regression model for mortality in the first six months after hip fracture is shown: malnutrition (HR: 2.61, 1.34–5.06), older age (70–76 years: HR: 2.5, 0.52–11.99; 77–82 years: HR: 4.25, 1.15–15.62; 83–99 years: HR: 3.82, 1.05–13.88) and a higher CCI score ≥ 3 (HR: 5.4, 1.53–19.12) were associated with higher risk of mortality.

**Table 2:** Binary logistic regression model for malnutrition among ED patients with hip fracture.

	OR	Lower 95% CI	Upper 95% CI	value of *p*
Intercept	5,79	0,78	42,80	0,087
Age (70–76 y)^a^	1,99	0,74	5,40	0,175
Age (77–82 y)	1,08	0,45	2,57	0,868
Age (83–99 y)	1,78	0,77	4,11	0,179
Gender (men)^b^	1,02	0,53	1,96	0,946
BMI (kg/m^2^)	0,89	0,83	0,96	0,001
Cognitive impairment (moderate and severe)^c^	1,34	0,67	2,67	0,409
CCI (= 1)^d^	0,98	0,40	2,42	0,963
CCI (= 2)	1,51	0,59	3,86	0,387
CCI (≥ 3)	1,39	0,61	3,18	0,440
Reduced physical activity (1x/months or less)^e^	0,28	0,14	0,58	0,001
Depression (PHQ-4 ≥ 6)^f^	1,20	0,49	2,97	0,695

Reference categories: ^a^ 50–69 y, ^b^ women, ^c^ no cognitive impairment, ^d^ CCI = 0, ^e^ physical activity 1x / week or more, ^f^ PHQ-4 score < 6 (no depressive symptoms).

ED, Emergency Department. CI, Confidence Interval. OR, Odds Ratio. BMI, body mass index. CCI, Charlson Comorbidity Index. PHQ-4, Patient Health Questionnaire-4.

**Table 3:** Cox proportional hazards model of six-month mortality among patients with hip fracture.

	HR	Lower 95% CI	Upper 95% CI	value of *p*
*Unadjusted Model*				
Malnutrition^a^	3.08	1.61	5.91	0.001
*Adjusted Model*				
Malnutrition^a^	2.61	1.34	5.06	0.006
Age (70–76 y)^b^	2.50	0.52	11.99	0.242
Age (77–82 y)	4.25	1.15	15.62	0.031
Age (83–99 y)	3.82	1.05	13.88	0.042
Gender (men)^c^	0.93	0.46	1.91	0.846
CCI (= 1)^d^	2.48	0.62	9.95	0.193
CCI (= 2)	1.91	0.42	8.56	0.388
CCI (≥ 3)	5.40	1.53	19.12	0.011

Reference categories: ^a^ normal nutritional status, ^b^ 50–69 y, ^c^ women, ^d^ CCI = 0.

ED, Emergency Department. CI, Confidence Interval. HR, Hazard Ratio. CCI, Charlson Comorbidity Index.

## Discussion

In this study of ED patients with hip fracture over 50 years of age, the risk of malnutrition was observed in every fourth patient. Malnutrition was associated with a higher risk of mortality during the first six months after adjusting for age, gender, and the burden of comorbidities. Several studies have shown an association of the mortality risk in patients with hip fractures with various patient and health system factors. However, few studies have investigated the underlying mechanisms that influence mortality in patients with hip fracture (35). A systematic review by Xu and colleagues identified the following predictors for mortality in patients with hip fracture: medical factors (presence of concomitant diseases, sarcopenia), surgical factors (including delay of surgery, e.g., > 48 h), type of fracture, socioeconomic factors (age, gender and ethnicity), and system factors (centers with lower case volume) (35). Malnutrition in old patients with hip fracture has at least three different dimensions: Malnutrition is a risk factor for hip fracture (36), it is associated with reduced functional capacity and worse recovery after hospital discharge (37), and, as shown in our study, it is associated with a higher mortality rate.

The 25.3% prevalence of malnutrition risk is within the range of prevalence rates found in studies with similar patient populations and clinical settings (4). Previous studies reported that the prevalence of malnourished patients with hip fracture has increased and ranges from about 7 to 26% (1). Compared to our study, the recently published Irish OPTI-MEND study showed in secondary analyses a lower prevalence of malnutrition (7.6%) in older ED patients, using the validated Mini Nutritional Assessment Short-Form (MNA-SF) screening tool. However, they found a higher number of ED patients with ED patients with malnutrition had lower MTS triage levels and had a lower risk of adverse health outcomes (38). This may partly be explained by our cohort, since hip fractures are associated with malnutrition, and these patients are typically more vulnerable or frail compared to older ED patients overall. The OPTI-MEND data revealed that at 30 days after ED, malnourished patients had a higher readmission rate, increased functional limitations and lower quality of life (38).

Differences in prevalence of malnutrition in studies can also be explained by the usage of different tools for the assessment of malnutrition. A recent secondary data analysis including 11 European and one New Zealand study with over 5,000 older adults showed higher malnutrition rates in adults >80 years of age, in women, and in people with one or more morbidities, with varying prevalence rates depending on geographic location and the tools used (39). Despite numerous publications and international discussions, there was no generally accepted definition of malnutrition until 2019, the time our study was conducted. Today, the concept of the Global Leadership Initiative on Malnutrition (GLIM) defines diagnostic criteria of malnutrition in all clinical settings, initiated by four world-leading clinical nutrition societies: the American Society for Parenteral and Enteral Nutrition, the European Society of Clinical Nutrition and Metabolism, Federación Latinoamericana de Terapia Nutritional, Nutrición Clínica y Metabolismo, and The Parenteral and Enteral Nutrition Society of Asia (40). The GLIM concept includes phenotypic parameters such as weight loss (> 5% in the last six months or > 10% beyond six months), low BMI (< 70 years: < 20 kg/m^2^; > 70 years: < 22 kg/m^2^), and reduced muscle mass as well as etiologic parameters such as reduced food intake, food reduction for more than 2 weeks, or any chronic gastrointestinal diseases and inflammation (40). In our study, one of the phenotypic criteria (involuntary weight loss) and one of the etiologic criteria (reduced food intake) were considered through the validated SNAQ instrument. Our study showed no difference between patients with and without malnutrition when assessing important ED routine parameters such as vital signs and triage levels, so malnourished patients may remain unnoticed without targeted screening.

Causes of malnutrition are diseases, aging processes, and lifestyle factors; the interaction between these factors is known. Patients screened for the risk of malnutrition in our study were older, had a reduced BMI, a higher number of medications per day, and more often experienced complications during their hospital stay. These malnourished patients were also more likely to be reduced in their physical activity status before hip fracture. Furthermore, patients with malnutrition risk were more likely to have a higher depression score. The regression analysis revealed lower BMI and self-reported lower physical activity as relevant factors associated with malnutrition risk in older ED patients with hip fracture. However, malnutrition in obese patients with higher BMIs is rarely recognized or completely missed since their fat mass masks the underlying muscle breakdown, and weight loss due to malnutrition is not detected (41). Although malnutrition was predominantly associated with a low BMI in our study, 12.1% of the cohort were obese (BMI ≥ 30.0 kg/m^2^). A higher BMI was recently found to be a nutritional risk factor for malnutrition in older adults (42) and the geriatric syndrome sarcopenic obesity is equally important in this context (43).

Data conflicting with our results have been published concerning comorbidity risk factors for negative nutritional outcomes (7), whereas in our study these parameters were not associated with malnutrition risk. We suspect that these differences to previous studies can be explained by the heterogeneity of older adults and their health conditions (44). Identifying the determinants of malnutrition is critical to effectively tackling the issue, but the complex etiology of malnutrition is still not completely understood. However, predicting nutritional deficiencies in older ED patients with hip fracture could reduce the negative impact on the patient’s functional status and quality of life (1) as well as reduce the health costs and rate of adverse events (45).

The study has clear strengths. First, as a health services research study, it aimed to be inclusive and patient-centered: we tried to include all patients affected by a hip fracture including cognitively impaired patients that were unable to participate in the interview themselves as well as hard to reach patients such as people from community shelters, nursing homes, and patients with a migration background. Second, the use of the short SNAQ questionnaire can be seen as another strength. With its three question units it can elicit malnutrition in times of high clinical workload through a quick and concrete evaluation. The study has a few limitations. The integrative real-life approach leads to a comparatively heterogeneous sample. Our results cannot be generalized to other population groups since the data come from an inner-city neighborhood of a major German city with specific living and healthcare conditions. Furthermore, body weight and height were self-reported resulting in a potential recall bias. The anthropometric data from our EMAAge cohort cannot easily be compared with other studies using medical measurement instruments.

## Conclusion

Malnutrition is an important risk factor for death after hip fracture. However, to date, a systematic screening for malnutrition is not performed in all patients with hip fractures or other geriatric indications. Identification of particularly vulnerable, older, malnourished patients in the ED or the subsequent in-patient stay could prevent negative outcomes and lead to the initiation of a patient-centered care approach including nutritional therapy. A recently implemented national guideline for the treatment of hip fracture patients defines new standards of care (46). This includes a systematic screening for geriatric syndromes and the involvement of geriatricians which has been recommended and evaluated internationally (47). The potential of this approach to meet the risks of malnutrition needs to be analyzed in future studies.

## Data availability statement

The raw data supporting the conclusions of this article will be made available by the authors, without undue reservation for scientific reasons.

## Ethics statement

The studies involving human participants were reviewed and approved by Ethics committee of Charité –Universitätsmedizin Berlin. The patients/participants provided their written informed consent to participate in this study.

## Author contributions

MM is initiator of the EMANet research network, principal investigator, and speaker. LS is a co-speaker of EMANet. LS and JD designed the EMAAge study. JD processed the data and performed basic analyses. KF designed the research question and drafted the first version of the manuscript. KF, JD, and MP created the statistical plan. MP did the multiple imputations and the corresponding analyses. KF and JD gave the interpretation of the data. DR, MM, TL, KN, and UM-W gave critical advice. All authors contributed to the article and approved the submitted version.

## Funding

The EMANet project is supported by the Federal Ministry of Education and Research (Bundesministerium für Bildung und Forschung -BMBF, grant number: 01GY1604). The sponsor is not involved in the design and conduct of the study, data collection, analysis, and interpretation of data or in writing this manuscript. We acknowledge financial support from the Open Access Publication Fund of Charité – Universitätsmedizin Berlin and the German Research Foundation (DFG).

## Conflict of interest

The authors declare that the research was conducted in the absence of any commercial or financial relationships that could be construed as a potential conflict of interest.

## Publisher’s note

All claims expressed in this article are solely those of the authors and do not necessarily represent those of their affiliated organizations, or those of the publisher, the editors and the reviewers. Any product that may be evaluated in this article, or claim that may be made by its manufacturer, is not guaranteed or endorsed by the publisher.

## Abbreviations

BMI: Body mass index
CCI: Charlson Cormorbidity Index
ED: Emergency department
GCS: Glasgow Coma Scale
GLIM: Global Leadership Initiative on Malnutrition
LOS: Length of hospital stay
MTS: Manchester Triage System
MNA-SF: Mini Nutritional Assessment Short-Form
PHQ-4: Patient Health Questionnaire 4
ONS: Oral nutritional supplements
SNAQ: Short Nutritional Assessment Questionnaire
